# On Vinum Sem. Colchici Opiate in Gonorrhea

**Published:** 1849-08

**Authors:** 


*On Vinum Sem. Colchici Opiate in Gonorrhea.* By Dr. Froinus.—Dr. Eisenmann, in a communication to the "Wochenschrift," in 1847, demonstrated the great utility of the Vin. sem. colch. (V. s. c, ʒiij.; tr. opii, ℈½), in doses of from 25 to 30 drops three or four times a day, in the treatment of both male and female gonorrhea.—Without denying that it may be sometimes desirable to precede its use by purgatives or oleaginous fluids, he had himself found it applicable in all stages of the disease, effecting a cure, upon an average, in about seven days. In the present paper, 10 additional cases are related of its successful employment in various stages of the affection; and reference made to some 50 others, in which it proved as satisfactory in the results. It is not only useful in infectious gonorrhea, but in discharges from the mucous membranes from other causes.—*Ohio Med. and Surgical Journal*, from *Casper's Wochenschrift.*

